# Tandem catalysis of ring-closing metathesis/atom transfer radical reactions with homobimetallic ruthenium–arene complexes

**DOI:** 10.3762/bjoc.6.133

**Published:** 2010-12-08

**Authors:** Yannick Borguet, Xavier Sauvage, Guillermo Zaragoza, Albert Demonceau, Lionel Delaude

**Affiliations:** 1 Laboratory of Macromolecular Chemistry and Organic Catalysis, Institut de Chimie (B6a), Université de Liège, Sart-Tilman par 4000 Liège, Belgium; 2 Unidade de Raios X, Edificio CACTUS, Universidade de Santiago de Compostela, Campus Vida, 15782 Santiago de Compostela, Spain

**Keywords:** Grubbs catalyst, indenylidene ligands, Kharasch reaction, microwave heating, olefin metathesis

## Abstract

The tandem catalysis of ring-closing metathesis/atom transfer radical reactions was investigated with the homobimetallic ruthenium–indenylidene complex [(*p*-cymene)Ru(μ-Cl)_3_RuCl(3-phenyl-1-indenylidene)(PCy_3_)] (**1**) to generate active species in situ. The two catalytic processes were first carried out independently in a case study before the whole sequence was optimized and applied to the synthesis of several polyhalogenated bicyclic γ-lactams and lactones from α,ω-diene substrates bearing trihaloacetamide or trichloroacetate functionalities. The individual steps were carefully monitored by ^1^H and ^31^P NMR spectroscopies in order to understand the intimate details of the catalytic cycles. Polyhalogenated substrates and the ethylene released upon metathesis induced the clean transformation of catalyst precursor **1** into the Ru(II)–Ru(III) mixed-valence compound [(*p*-cymene)Ru(μ-Cl)_3_RuCl_2_(PCy_3_)], which was found to be an efficient promoter for atom transfer radical reactions under the adopted experimental conditions.

## Introduction

**Scheme 1:**
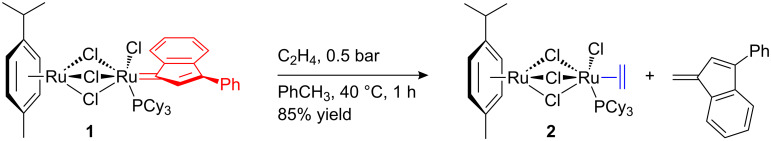
Reaction of homobimetallic ruthenium–indenylidene complex **1** with ethylene.

During the course of our investigations on homobimetallic ruthenium–arene complexes, we found that the indenylidene compound [(*p*-cymene)Ru(μ-Cl)_3_RuCl(3-phenyl-1-indenylidene)(PCy_3_)] (**1**) was a very efficient promoter for the ring-closing metathesis (RCM) of diethyl 2,2-diallylmalonate [1]. Contrastingly, this catalyst precursor was almost inactive in the self-metathesis of styrene, as stilbene formation leveled off after a few minutes without going past the 10% threshold. We attributed this negative result to a rapid degradation of the active species via a bimolecular pathway leading to the ethylene complex [(*p*-cymene)Ru(μ-Cl)_3_RuCl(η^2^-C_2_H_4_)(PCy_3_)] (**2**). Support in favor of this hypothesis came from the observation that complex **1** reacted quantitatively with ethylene at 40 °C to afford product **2** [1], which is completely devoid of metathetical activity (Scheme 1) [2]. Moreover, early work from Grubbs and co-workers had shown that bimetallic ruthenium–methylidene or ethylidene complexes decomposed rapidly to afford an unidentified ruthenium–ethylene species [3]. We were able to isolate and characterize this product, which turned out to be complex **2** [1]. The synthesis of this compound was first reported in 2005 by Severin et al. who successfully used it as a catalyst for atom transfer radical addition (ATRA) and cyclization (ATRC) reactions [4–5]. In 2007, we further extended its application field to the related process of atom transfer radical polymerization (ATRP) [2].

**Scheme 2:**
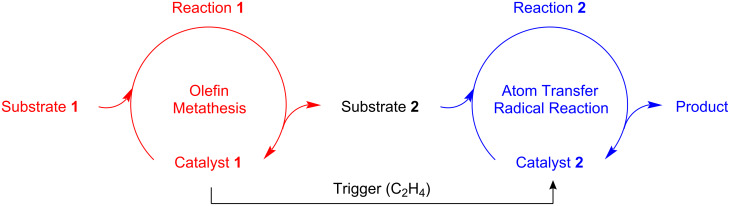
Schematic illustration of tandem assisted catalysis with complexes **1** and **2**.

Because the transformation of complex **1** into compound **2** occurs seamlessly in the presence of ethylene, which is a byproduct of many metathesis reactions, we reasoned that it could serve to trigger a change in mechanism, thereby allowing us to perform two consecutive catalytic cycles in a single procedure (Scheme 2). This process, known as assisted tandem catalysis [6], presents significant advantages over multistep synthesis for increasing molecular complexity, particularly in terms of time- and cost-savings, atom economy, environmental friendliness, or applicability to diversity-oriented high-throughput synthesis [7–10]. The monometallic ruthenium–benzylidene complex [RuCl_2_(=CHPh)(PCy_3_)_2_] (**3**) and its second- or even third-generation analogues developed by Grubbs and co-workers are prominent examples of catalyst precursors that were applied to olefin metathesis in tandem with ATRA [11], ATRC [11–14], ATRP [15–18], cyclopropanation [19], dihydroxylation [20], hydrogenation [21–23], hydrovinylation [24], isomerization [25–28], oxidation [29], or Wittig reactions [30], to name just a few [31].

In this contribution, we investigate the tandem catalysis of RCM/ATRC reactions with homobimetallic ruthenium–indenylidene complex **1** to generate active species in situ. The two catalytic processes were first carried out independently in a case study before the whole sequence was optimized and applied to the synthesis of several polyhalogenated bicyclic γ-lactams and lactones.

## Results and Discussion

**Scheme 3:**
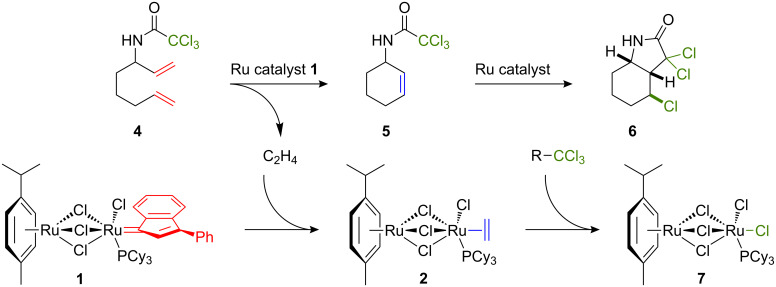
Tandem RCM/ATRC of 2,2,2-trichloro-*N*-(octa-1,7-dien-3-yl)acetamide (**4**) catalyzed by complex **1**.

2,2,2-Trichloro-*N*-(octa-1,7-dien-3-yl)acetamide (**4**) was chosen as a model substrate to begin our investigations (Scheme 3). The RCM of this functionalized α,ω-diene was carried out in toluene-*d*_8_ (0.2 M) at 30 °C in the presence of 5 mol % of catalyst precursor **1** and monitored by ^1^H NMR spectroscopy. Under these conditions, ring closure took place readily and a full conversion of the substrate into its cyclohexene derivative **5** was achieved within 20 minutes. At this temperature, the second step of ATRC did not occur. Previous work had shown that a significant thermal activation was required to perform the radical cyclization of this cyclohexenyl trichloroacetamide, presumably due to the unfavorable disposition of the trichloromethyl unit and the endocyclic double bond in the most stable rotamer of the amido group [11]. Hence, this preliminary experiment allowed us to determine the nature of the catalytic species present in the reaction mixture after the metathesis step. No meaningful information could be obtained by ^31^P NMR spectroscopy even when acquisition was prolonged overnight to compensate for the low catalyst concentration in the sample. Visual inspection of the NMR tube revealed, however, the formation of a phosphorus-containing precipitate. Suitable crystals for X-ray diffraction analysis were obtained by repeating the RCM experiment on a larger scale in toluene at room temperature. Their structure was solved and assigned to the paramagnetic complex [(*p*-cymene)Ru(μ-Cl)_3_RuCl_2_(PCy_3_)] (**7**). This mixed valence Ru(II)–Ru(III) compound had already been isolated and fully characterized by Severin and co-workers when they investigated the reaction of ethylene complex **2** with carbon tetrachloride in toluene [4]. Yet, differences between the molecular structures obtained by the Swiss team and our group indicate that complex **7** can adopt various crystalline structures (see Supporting Information File 1 for more details on crystal structures and Supporting Information File 2 for X-ray crystal data).

The clean transformation of catalyst precursor **1** into compound **7** induced by polyhalogenated substrates is in line with the general mechanism postulated for ruthenium-catalyzed atom transfer radical reactions, as it involves a reversible oxidation of the metal center [32–33]. Under the experimental conditions adopted for our study, conversion of indenylidene precursor **1** into labile ethylene complex **2** probably occurred rapidly upon release of ethylene in the reaction mixture by the RCM of substrate **4** (Scheme 3). The low concentration of compound **2** in solution prevented, however, its instantaneous detection by NMR spectroscopy. When a longer acquisition time was applied, only oxidized product **7** was obtained. It should be noted that when the Grubbs first-generation catalyst **3** was allowed to react with substrate **4** for 2 h in toluene-*d*_8_ at room temperature, the RCM product **5** was also formed quantitatively. In this case, however, ^31^P NMR analysis of the reaction mixture revealed the presence of at least five different ruthenium–phosphine species in solution. Unless all these species are able to promote the ATRC reaction, the catalytic switch required to complete the tandem process should therefore be far less efficient with monometallic benzylidene complex **3** than with bimetallic indenylidene precursor **1**.

Next, we investigated separately the ATRC of 2,2,2-trichloro-*N*-(cyclohex-2-en-1-yl)acetamide (**5**) with different catalyst precursors. The starting material employed in these experiments was prepared by trichloroacetylation of 2-cyclohexenol with CCl_3_COCl in the presence of Et_3_N [34]. This procedure guaranteed the absence of any residual metal catalyst coming from the RCM reaction. A solution of 2-cyclohexenyl trichloroacetamide **5** in toluene was heated for 2 h at 160 °C in the presence of various ruthenium initiators (1 mol %). Conversion into racemic product **6** was then determined by GC analysis of the reaction mixture (Scheme 3 and Table 1). Previous work from the group of Itoh et al. had already established that the ruthenium-catalyzed cyclization of *N*-allyl trichloroacetamides proceeded diastereoselectively, and a mechanism accounting for the formation of a *cis*-fused bicyclic system was proposed [35]. Nuclear Overhauser effects also indicated that the angular H-3a and the CHCl H-4 protons were *trans* to each other.

**Table 1:** ATRC of 2,2,2-trichloro-*N*-(cyclohex-2-en-1-yl)acetamide (**5**) catalyzed by various ruthenium complexes.^a^

Entry	Ru cat.	Conversion (%)^b^

1	–	0
2	**1**	94
3	**2**	98
4	**3**	55
5	**7**	97

^a^Experimental conditions: substrate (0.2 mmol), catalyst (2 μmol), toluene (1 mL) in a sealed tube under Ar for 2 h at 160 °C. ^b^Determined by GC with *n*-dodecane as internal standard.

Because radical reactions may occur spontaneously at high temperature, we first carried out a blank test in the absence of an initiator (Table 1, entry 1). This experiment confirmed the necessity of mediating the transformation of **5** into **6** with a transition metal complex. Unlike the Grubbs benzylidene catalyst **3**, bimetallic compound **1** was an efficient catalyst precursor for this reaction (Table 1, entries 2 and 4). Opstal and Verpoort had already established that monometallic ruthenium–indenylidene complexes were able to promote the ATRA and ATRP of vinyl monomers [36–37]. In a tandem RCM/ATRC process, it is, however, very unlikely for the indenylidene species to remain unaltered in solution after the metathesis step. Indeed, during the course of our investigations on the RCM of various α,ω-dienes catalyzed by complex **1**, ^31^P NMR monitoring of the reaction media always showed a rapid disappearance of the signal originating from this precatalyst, and its replacement by a new singlet at ca. 40.5 ppm due to the ethylene complex **2**. As expected, this compound was highly suitable for catalyzing the ATRC of **5** (Table 1, entry 3). To our great satisfaction, oxidation product **7** was equally active under the experimental conditions adopted for this cyclization and did not require any co-catalyst (Table 1, entry 5). This result contrasts with previous observations from Severin and co-workers, who found that the presence of a radical initiator or a reducing agent (typically Mg) was mandatory to activate complex **7** for ATRA and ATRC reactions at room temperature [5]. At 160 °C, a reduction of the mixed Ru(II)–Ru(III) compound probably takes place under the sole influence of radicals generated via thermal dissociation of the substrate.

In order to complete the full sequence of RCM and ATRC reactions, we carried out a third series of catalytic tests based on literature procedures developed for this type of tandem catalysis [11,13–14]. These experiments were conducted on a preparative microscale in sealed tubes under inert atmosphere. Substrate **4** and complex **1** were dissolved in toluene. A color change from red to orange occurred within a few minutes, which indicated the formation of metathetically active species. Stirring was prolonged for 2 h at 25 °C. The vessel was then heated in an oil bath to trigger the ruthenium-catalyzed cyclization of intermediate **5** into 3,3,4-trichlorohexahydro-1*H*-indol-2(3*H*)-one (**6**) (Scheme 3). This final product was isolated by column chromatography. Its identity and purity were confirmed by ^1^H and ^13^C NMR analyses. Table 2 summarizes the results of these experiments.

**Table 2:** Tandem RCM/ATRC of 2,2,2-trichloro-*N*-(octa-1,7-dien-3-yl)acetamide (**4**).

Entry	Catalyst precursor	Exp. conditions for RCM	Exp. conditions for ATRC^a^	Isolated yield of product 6 (%)

1	**1** (5 mol %)	25 °C, 2 h	Δ, 160 °C, 2 h	71
2	**3** (5 mol %)	25 °C, 2 h	Δ, 160 °C, 2 h	61
3	**1** (5 mol %)	40 °C, 2 h	Δ, 110 °C, 2 h	0^b^
4	**1** (1 mol %)	25 °C, 30 min	Δ, 160 °C, 2 h	76
5	**3** (1 mol %)	25 °C, 2 h	Δ, 160 °C, 2 h	20
6	**1** (0.5 mol %)	–	Δ, 160 °C, 2 h	0^b^
7	**1** (1 mol %)	–	μw, 160 °C, 40 min	73

^a^Δ: conductive heating in an oil bath, μw: microwave heating in a monomodal reactor. ^b^Only RCM product **5** was present.

When a 5 mol % catalyst loading was employed and the ATRC reaction was allowed to proceed for 2 h at 160 °C, bicyclic lactam **6** was isolated in 71% yield (Table 2, entry 1). We were pleased to note that homobimetallic complex **1** slightly outperformed the Grubbs first-generation catalyst **3**, which led to a 61% yield under identical conditions (Table 2, entry 2). We tried to further optimize the catalytic process by reducing the reaction temperature and the catalyst loading. Performing the second step at 110 °C completely inhibited the cyclization as evidenced by GC analysis, which revealed a complete conversion of substrate **4** into intermediate **5**, but did not show any sign of product **6** formation (Table 2, entry 3). On the other hand, it was possible to accomplish the dual catalysis at 160 °C with only 1 mol % of catalyst precursor **1** (Table 2, entry 4). The slight increase in isolated yield compared to run #1 should not be over interpreted. It probably reflects the systematic errors in the weighing of the reagents and in the chromatographic purification of the product formed. A control experiment carried out with [RuCl_2_(=CHPh)(PCy_3_)_2_] **3** confirmed the superiority of the bimetallic system under these conditions (Table 2, entry 5). Attempts to further decrease the molar ratio of complex **1** remained unsuccessful (see Table 2, entry 6 for a representative example). Finally, we were able to significantly shorten and simplify the whole process through the use of a monomodal microwave reactor (Table 2, entry 7). Such a device is becoming increasingly popular in organic synthesis and has already been used as a convenient heating source for numerous ruthenium-catalyzed reactions [38].

Because thermal degradation of the catalyst is likely to occur at the high temperature required to promote the ATRC of 2-cyclohexenyl trichloroacetamide **5**, we searched for alternative substrates that would allow us to perform the tandem reaction under less drastic conditions. We were guided in this endeavor by Snapper et al. who had shown that adding a benzyl or tosyl group to the amide functionality of compound **5** facilitated its radical cyclization mediated by complex **3**. Replacing the trichloroacetamide moiety with the corresponding tribromoacetamide unit was also found to enhance the Kharasch reactivity [11]. Thus, we synthesized four additional *N*-protected octadienyl trichloro- or tribromoacetamide substrates and we followed their transformation under the influence of bimetallic catalyst precursor **1** (see Supporting Information File 3 for details). As expected, *N*-benzyl trichloroacetamide **8** underwent the RCM/ATRC sequence at a lower temperature than its parent **4** (110 °C vs 160 °C), although the reaction time had to be extended in order to achieve a full conversion into bicyclic lactam **9** (Table 3, entry 1). The reaction of *N*-tosyl trichloroacetamide **10** proceeded faster, but followed a different course, as demonstrated by the isolation of a diastereomeric mixture of unsaturated bicyclic lactams **11** (Table 3, entry 2). The two products were separated by column chromatography. 2D NMR spectroscopy and mass spectrometry analyses confirmed that dehydrochlorination had occurred during the catalytic process. Further work is underway to rationalize this change of reaction path and to address all its stereochemical implications.

**Table 3:** Reactions of various octadienyl trichloro- or tribromoacetamide substrates catalyzed by complex **1**.^a^

Entry	Substrate	Product(s)	Conditions	Isolated yield (%)

1	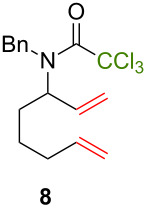	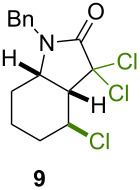	μw, 110 °C, 4 h	89%
2	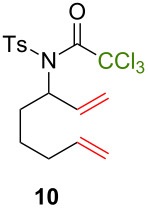	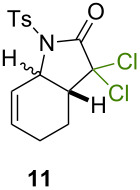	μw, 110 °C, 2 h	73%^b^
3	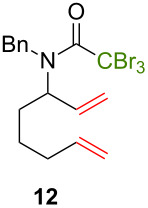	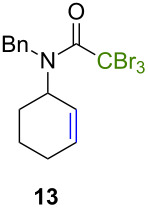	25 °C, 2.5 h	89%
4	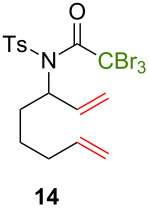	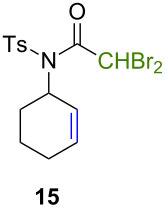	25 °C, 2.5 h	96%

^a^Experimental conditions: substrate (0.3 mmol), catalyst (3 μmol), and toluene (1.5 mL) stirred in a pressure vessel under Ar in a monomodal microwave reactor (μw, 110 °C) or in a thermostated oil bath (25 °C). ^b^Diastereomeric ratio: 44:56.

With the *N*-benzyl tribromoacetamide **12**, the RCM step proceeded swiftly at 25 °C, but ATRC did not occur at this temperature, in sharp contrast with previous results obtained by Snapper et al. with catalyst precursor **3** [11]. Work-up afforded only cyclohexenyl tribromoacetamide **13** in high yield (Table 3, entry 3). Attempts to promote the cyclization of this compound at higher temperatures led to a complex mixture of uncharacterized products. A final experiment carried out with the *N*-tosyl tribromoacetamide **14** afforded quantitatively the cyclohexenyl dibromoacetamide **15** (Table 3, entry 4). We suspected that the high Kharasch reactivity of the starting material or the intermediate RCM product caused a radical transfer to the solvent. However, performing the reaction in benzene or dichloromethane instead of toluene led to similar outcomes. Adding a larger amount of complex **1** (5 mol %) to substrates **12** and **14** did not seem to improve the ATRC step.

**Scheme 4:**
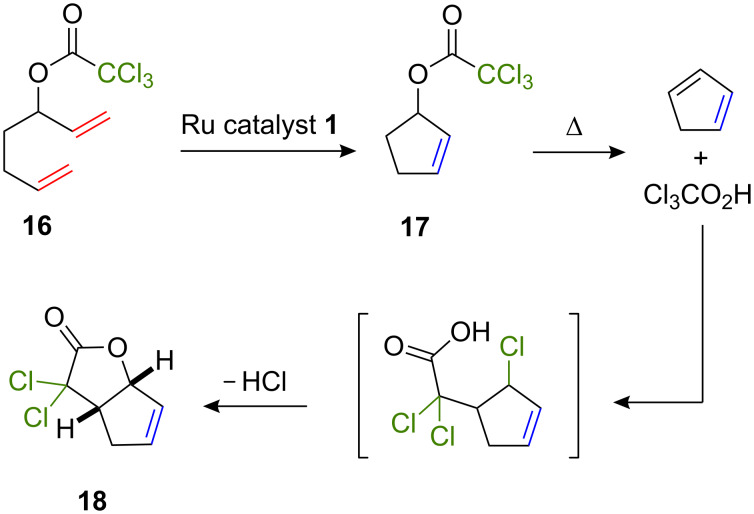
Ruthenium catalyzed transformation of substrate **16**.

To complement the data acquired with trihaloacetamide starting materials, we also investigated the transformation of hepta-1,6-dien-3-yl 2,2,2-trichloroacetate **16** in the presence of ruthenium–indenylidene catalyst precursor **1** (5 mol %). A preliminary experiment was carried out in toluene-*d*_8_ and monitored by ^1^H NMR spectroscopy. The RCM of the α,ω-diene occurred readily at 25 °C and a quantitative conversion into cyclopentene derivative **17** was achieved within 1 h (Scheme 4). The temperature was then raised to 60 °C in an attempt to initiate an ATRC reaction. Under these conditions, ^1^H NMR analysis unambiguously revealed the formation of cyclopentadiene instead of the expected bicyclic lactone. The decomposition of cyclopentenyl trichloroacetate **17** into cyclopentadiene and trichloroacetic acid was already observed by Quayle et al. under similar conditions [13–14]. These authors successfully trapped the diene via a Diels–Alder reaction with maleic anhydride. They also reported that a heterobimetallic catalytic system derived from the Grubbs second-generation complex [RuCl_2_(=CHPh)(SIMes)(PCy_3_)], CuCl, and dHbipy (SIMes is 1,3-dimesitylimidazolin-2-ylidene, dHbipy is 4,4'-di-*n*-heptyl-2,2'-bipyridine) was able to promote the ATRA of trichloroacetic acid onto cyclopentadiene followed by a lactonization into 3,3-dichloro-3,3a,4,6a-tetrahydro-2*H*-cyclopenta[*b*]furan-2-one (**18**). These results prompted us to examine the cascade RCM/decomposition/ATRA/lactonization of substrate **16** into dichloro compound **18** with homobimetallic complex **1** under mild thermolysis conditions. Thus, the substrate and the catalyst precursor (5 mol %) were dissolved in toluene and heated for 1 h at 80 °C under microwave irradiation. Under these conditions, product **18** was isolated in 52% yield after chromatographic purification. ^1^H NMR data matched those reported for a sample known to possess a *cis* stereochemistry for its bridgehead hydrogens [39].

## Conclusion

In this study, we have demonstrated that homobimetallic ruthenium–indenylidene complex **1** is a suitable catalyst precursor for the tandem RCM/ATRC of polyhalogenated α,ω-dienes **4** and **8** into the corresponding bicyclic γ-lactam derivatives. A more complex cascade sequence involving RCM and ATRA reactions afforded γ-lactone **18** starting from acyclic unsaturated ester **16**. The individual steps were carefully monitored by ^1^H and ^31^P NMR spectroscopies in order to understand the intimate details of the catalytic cycles. The RCM of model substrate **4** into cyclohexenyl trichloroacetamide **5** was accompanied by a clean transformation of complex **1** into mixed-valence bimetallic scaffold **7**. This well-defined compound was an efficient promoter for the ATRC of intermediate **5** into the final product **6**.

## Supporting Information

Full experimental procedures and spectral data for the new compounds, detailed crystallographic analysis of [(*p*-cymene)Ru(μ-Cl)_3_RuCl_2_(PCy_3_)] (**7**), and a cif file with crystallographic data for complex **7**.

File 1 Experimental procedures and spectral data.

File 2 Detailed crystallographic analysis of complex **7**.

File 3 X-ray crystal data for complex **7**.
