# Supplementary material for: Tandem catalysis of ring-closing metathesis/atom transfer radical reactions with homobimetallic ruthenium–arene complexes
Source: Beilstein J Org Chem. 2010 Dec 8;6:1167–73. doi: 10.3762/bjoc.6.133 (PMC3002019; doi:10.3762/bjoc.6.133)
Supplement: File 1 — Experimental procedures and spectral data. [file Beilstein_J_Org_Chem-06-1167-s001.pdf]

Supporting Information

for

Tandem catalysis of ring-closing metathesis/ atom transfer radical reactions with homobimetallic ruthenium-arene complexes

Yannick Borguet[1], Xavier Sauvage[1], Guillermo Zaragoza[2], Albert Demonceau[1] and Lionel Delaude[*1]

Address:
[1]Laboratory of Macromolecular Chemistry and Organic Catalysis, Institut de Chimie (B6a), Université de Liège, Sart-Tilman par 4000 Liège, Belgium and
[2]Unidade de Raios X, Edificio CACTUS, Universidade de Santiago de Compostela, Campus Vida, 15782 Santiago de Compostela, Spain

Email:
Lionel Delaude - l.delaude@ulg.ac.be

* Corresponding author

Supporting Information File 1
Experimental procedures and spectral data

Table of content

1. General information S1-2

2. Starting materials.. S1-3

*2.1. Synthesis of octa-1,7-dien-3-amine (**A**)* *S1-3*

*2.2. Synthesis of N-benzylocta-1,7-dien-3-amine (**B**)*... *S1-4*

*2.3. Synthesis of 2,2,2-tribromo-N-(octa-1,7-dien-3-yl)-N-tosylacetamide (**14**)*.............. *S1-4*

3. Typical procedure for the synthesis of 3,3,4-trichlorohexahydro-1*H*-indol-2(3*H*)-one (**6**) S1-4

4. Reactions of various octadienyl trichloro- or tribromoacetamide substrates catalyzed by complex 1 .... S1-5

*4.1. Synthesis of (3aS,4S,7aS)-1-benzyl-3,3,4-trichlorohexahydro-1H-indol-2(3H)-one (**9**)* *S1-5*

*4.2. Synthesis of (3aR)-3,3-dichloro-1-tosyl-3,3a,4,5-tetrahydro-1H-indol-2(7aH)-one (**11**)* *S1-5*

*4.3. Synthesis of N-benzyl-2,2,2-tribromo-N-(cyclohex-2-en-1-yl)acetamide (**13**)* *S1-5*

*4.4. Synthesis of 2,2-dibromo-N-(cyclohex-2-en-1-yl)-N-tosylacetamide (**15**)* *S1-5*

5. Synthesis of 3,3-dichloro-3,3a,4,6a-tetrahydro-2*H*-cyclopenta[*b*]furan-2-one (**18**)......... S1-6

References S1-6

1. General information

All reactions were carried out under a dry argon atmosphere using standard Schlenk techniques. Solvents were distilled from appropriate drying agents and deoxygenated prior to use. A CEM Discover instrument was used for microwave-assisted syntheses. Silica gel 60 (60 Å nominal pore diameter, 0.063–0.2 mm particle size) supplied by Biosolve was used for column chromatography. Petroleum ether refers to the hydrocarbon fraction of bp 40–60 °C and was purchased from Labotec. ^{1}H, $^{13}C\{^{1}H\}$, and $^{31}P\{^{1}H\}$ NMR spectra were recorded with a Bruker DRX 400 or a Bruker Avance 250 spectrometer. Chemical shifts are listed in parts per million downfield from TMS and are referenced from the solvent residual peaks (^{1}H, ^{13}C) or external H_3PO_4 (^{31}P). Gas chromatography was carried out with a Varian 3900 instrument equipped with a flame ionization detector and a WCOT fused silica column (stationary phase: CP-Sil 5CB, column length: 15 m, inside diameter: 0.25 mm, outside diameter: 0.39 mm, film thickness: 0.25 μm). Mass spectra were recorded with a Waters Q-TOF Ultima spectrometer (ESI+ mode, source temperature: 100 °C, capillary voltage: 3000 V, RF lens intensity: 100, source pressure: 3.3 mbar).

2. Starting materials

Complexes **1** [1], **2** [2], **7** [2], and substrate **5** [3] were synthesized according to published procedures. The preparation of *N*-octadienyl tribromo- or trichloroacetamides **4**, **8**, **10**, **12**, and **14** was carried out according to Scheme S1-1, based on experimental procedures devised by Snapper *et al.* [4] or Singh and Han [5]. For the synthesis of trichloroacetyl ester **16**, the procedure outlined by Quayle and co-workers was adopted (Scheme S1-2) [6]. 2,7-Octadienol was obtained as a mixture of *cis* and *trans* isomers from TCI. All the other chemicals were purchased from Aldrich.

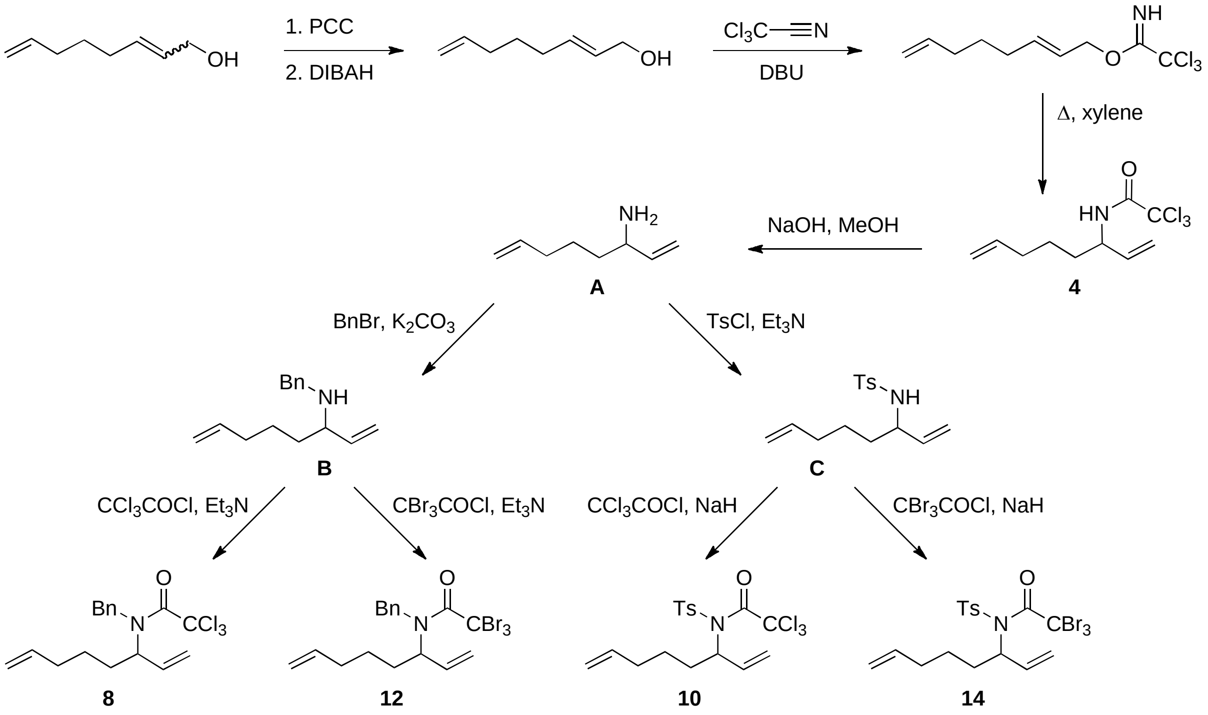

Scheme S1-1. Synthesis of the *N*-octadienyl tribromo- or trichloroacetamide substrates used in this work.

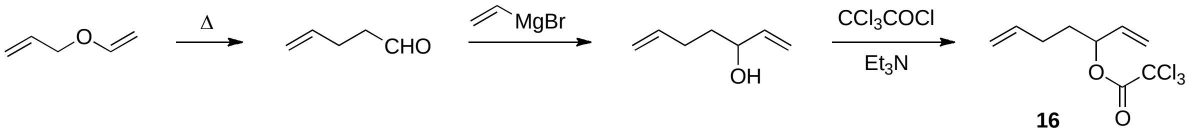

Scheme S1-2. Synthesis of hepta-1,6-dien-3-yl 2,2,2-trichloroacetate (**16**).

*2.1. Synthesis of octa-1,7-dien-3-amine (**A**)*

A 2 M aqueous solution of sodium hydroxide (63 mL, 126 mmol) was added dropwise to a solution of 2,2,2-trichloro-*N*-(octa-1,7-dien-3-yl)acetamide (**4**) (15.6 g, 58 mmol) in methanol (125 mL) under vigorous stirring. The reaction mixture was further stirred for 12 h at 50 °C. It was diluted with water (60 mL) and extracted with dichloromethane (3 × 150 mL). The organic phases were gathered and dried with anhydrous K_2CO_3. The solvents were removed on a rotary evaporator and the residue was purified by column chromatography on silica gel (eluent: petroleum ether/ethyl acetate 3/7 v/v) to afford the pure product as a yellow oil.

Yield: 6.8 g (94%). ^{1}H NMR (250 MHz, $CDCl_3$): δ = 1.34 (m, 6H), 1.99 (m, 2H), 3.19 (m, 1H), 4.84–5.05 (m, 4H), 5.60–5.80 (m, 2H) ppm. ^{13}C NMR (62.5 MHz, $CDCl_3$): δ = 143.4, 138.6, 114.5, 113.3, 54.4, 37.0, 33.6, 25.3 ppm.

*2.2. Synthesis of N-benzylocta-1,7-dien-3-amine (**B**)*

A solution of benzyl bromide (1.98 g, 11.6 mmol) in dry acetonitrile (10 mL) was added dropwise to a suspension of octa-1,7-dien-3-amine (**A**) (2 g, 16.5 mmol) and potassium carbonate (2.51 g, 18.2 mmol) in dry acetonitrile (50 mL). The reaction mixture was stirred for 24 h at room temperature. The inorganic salts were filtered off and the remaining solution was concentrated on a rotary evaporator. The residue was purified by column chromatography on silica gel (eluent: petroleum ether/ethyl acetate 9/1 v/v) to afford the pure product as a pale yellow oil. Yield: 2.31 g (92%). ^{1}H NMR (250 MHz, $CDCl_3$): δ = 7.30–7.20 (m, 5H), 5.85–5.53 (m, 2H), 5.15–4.90 (m, 4H), 3.81 (d, *J* = 13.2 Hz, 1H), 3.62 (d, *J* = 13.2 Hz, 1H), 3.00 (m, 1H), 2.02 (m, 2H), 1.55–1.32 (m, 4H) ppm. ^{13}C NMR (62.5 MHz, $CDCl_3$): δ = 141.3, 140.8, 138.7, 128.3, 128.2, 126.8, 116.0, 114.6, 61.1, 51.2, 35.2, 33.7, 25.2 ppm.

*2.3. Synthesis of 2,2,2-tribromo-N-(octa-1,7-dien-3-yl)-N-tosylacetamide (**14**)*

A solution of 4-methyl-*N*-(octa-1,7-dien-3-yl)benzenesulfonamide (**C**) (1 g, 3.6 mmol) in dry THF (15 mL) was added dropwise to a suspension of sodium hydride (0.489 g, 20.4 mmol) in dry THF (5 mL) cooled to 0 °C. The reaction mixture was brought back to room temperature and further stirred for 45 min before a solution of tribromoacetyl chloride (1.95 g, 10.7 mmol) in dry THF (5 mL) was added dropwise. The resulting solution was further stirred overnight at room temperature. The reaction mixture was quenched with a saturated aqueous solution of ammonium chloride (5 mL) and extracted with diethyl ether (3 × 50 mL). The organic phases were gathered and dried with anhydrous $MgSO_4$. The solvents were removed on a rotary evaporator and the residue was purified by column chromatography on silica gel (eluent: petroleum ether/ethyl acetate 9/1 v/v) to afford the pure product as a viscous oil. Yield: 1.84 g (92%). ^{1}H NMR (250 MHz, $CDCl_3$): δ = 1.30 (m, 2H), 1.79 (m, 2H), 2.00 (m, 2H), 2.45 (s, 3H), 4.51 (q, *J* = 7.2 Hz, 1H), 4.90 (m, 2H), 4.99 (m, 1H), 5.10 (m, 1H), 5.72 (m, 1H), 6.05 (m, 1H), 7.37 (d, *J* = 8.2 Hz, 2H), 7.81 (d, *J* = 8.2 Hz, 2H) ppm. ^{13}C NMR (62.5 MHz, $CDCl_3$): δ = 164.30, 145.95, 138.05, 135.28, 135.03, 130.24, 128.12, 119.28, 115.03, 64.07, 36.20, 33.12, 31.75, 25.98, 21.79 ppm.

3. Typical procedure for the synthesis of 3,3,4-trichlorohexahydro-1*H*-indol-2(3*H*)-one (6)

A 5 mL glass tube containing a magnetic stirring bar was loaded with a ruthenium complex (20 μmol). Air was expelled by three vacuum-argon cycles before a 0.2 M solution of 2,2,2-trichloro-*N*-(octa-1,7-dien-3-yl)acetamide (**4**) in dry and degassed toluene was added (2 mL, 0.4 mmol). The tube was sealed with an acetylene torch and the reaction mixture was stirred for 30 min at 25 °C and then for 2 h in an oil bath at 160 °C. Alternatively, the reaction was performed in a 10 mL pressure vial capped with a septum using a CEM Discover instrument with a maximum 150 W microwave power. After cooling to room temperature, the solvent was removed on a rotary evaporator. The residue was purified by column chromatography on silica gel (eluent: petroleum ether/ethyl acetate 98/2 v/v) to afford the pure product. ^{1}H and ^{13}C NMR spectra were identical to those reported in the literature [7].

4. Reactions of various octadienyl trichloro- or tribromoacetamide substrates catalyzed by complex 1

A 10 mL pressure vial containing a magnetic stirring bar and capped with a septum was loaded with complex **1** (2.8 mg, 3 μmol). Air was expelled by three vacuum-argon cycles before a substrate (0.3 mmol), and dry and degassed toluene (1.5 mL) were added. The reaction mixture was heated at 110 °C under stirring in a microwave reactor with a 150 W maximum power, or maintained at 25 °C under stirring in an oil bath. After cooling to room temperature, the solvent was removed on a rotary evaporator. The residue was purified by column chromatography on silica gel.

*4.1. Synthesis of (3aS,4S,7aS)-1-benzyl-3,3,4-trichlorohexahydro-1H-indol-2(3H)-one (**9**)*

Pale yellow oil (eluent: petroleum ether/diethyl ether 3/1 v/v). Yield: 88.5 mg (89%). ^{1}H and ^{13}C NMR spectra were identical to those reported in the literature [4].

*4.2. Synthesis of (3aR)-3,3-dichloro-1-tosyl-3,3a,4,5-tetrahydro-1H-indol-2(7aH)-one (**11**)*

Diastereomer 1: Pale yellow oil (eluent: petroleum ether/ethyl acetate 90/10 v/v). Yield: 35.0 mg (32%). ^{1}H NMR (400 MHz, $CDCl_3$): δ = 7.90 (d, *J* = 8.3 Hz, 2H), 7.33 (d, *J* = 8.3 Hz, 2H), 6.39 (m, 1H), 6.16 (m, 1H), 4.74 (m, 1H), 2.79 (m, 1H), 2.43 (s, 3H), 2.29–1.99 (m, 2H), 1.35–0.74 (m, 2H) ppm. ^{13}C NMR (101 MHz, $CDCl_3$): δ = 164.48, 145.84, 134.32, 134.23, 129.80, 128.55, 122.58, 85.17, 54.91, 47.97, 29.81, 23.93, 21.87 ppm. ESI MS: *m/z* = 360.064 ($C_{15}H_{15}SO_3Cl_2N$: 360.023).

Diastereomer 2: White solid (eluent: petroleum ether/ethyl acetate 70/30 v/v). Yield: 45.1 mg (41%). ^{1}H NMR (400 MHz, $CDCl_3$): δ = 7.85 (d, *J* = 8.2 Hz, 2H), 7.28 (d, *J* = 8.2 Hz, 2H), 6.36–6.18 (m, 1H), 6.08–5.95 (m, 1H), 5.24 (m, 1H), 2.87 (m, 1H), 2.41 (s, 3H), 2.34–2.04 (m, 2H), 1.38–0.98 (m, 2H) ppm. ^{13}C NMR (101 MHz, $CDCl_3$): δ = 165.63, 143.95, 137.59, 137.39, 129.40, 127.55, 120.41, 86.19, 79.65, 50.65, 23.88, 21.69, 21.63 ppm. ESI MS: *m/z* = 360.072 ($C_{15}H_{15}SO_3Cl_2N$: 360.023)

*4.3. Synthesis of N-benzyl-2,2,2-tribromo-N-(cyclohex-2-en-1-yl)acetamide (**13**)*

Pale yellow oil (eluent: petroleum ether/ethyl acetate 95/5 v/v). Yield: 125.0 mg (89%). ^{1}H NMR (400 MHz, $CDCl_3$): δ = 7.46–7.29 (m, 2H), 7.29–7.16 (m, 3H), 5.99 (s, 1H), 5.70 (d, *J* = 9.8 Hz, 1H), 5.45 (s, 1H), 4.79 (d, *J* = 15.7 Hz, 1H), 4.37 (d, *J* = 15.7 Hz, 1H), 2.27–2.12 (m, 1H), 2.12–1.92 (m, 2H), 1.92–1.73 (m, 1H), 1.73–1.50 (m, 2H) ppm. ^{13}C NMR (101 MHz, $CDCl_3$): δ = 160.56, 138.02, 133.15, 128.61, 127.21, 126.93, 126.18, 58.24, 49.07, 37.07, 27.58, 24.63, 21.58 ppm.

*4.4. Synthesis of 2,2-dibromo-N-(cyclohex-2-en-1-yl)-N-tosylacetamide (**15**)*

Pale yellow oil (eluent: petroleum ether/ethyl acetate 90/10 v/v). Yield: 130.0 mg (96%). ^{1}H NMR (400 MHz, $CDCl_3$): δ = 7.84 (d, *J* = 8.2 Hz, 2H), 7.36 (d, *J* = 8.2 Hz, 2H), 6.85 (s, 1H), 5.92–5.78 (m, 1H), 5.38 (d, *J* = 10.2 Hz, 1H), 4.85 (m, 1H), 2.44 (s, 3H), 2.15–1.81 (m, 6H), 1.72–1.52 (m, 1H) ppm. ^{13}C NMR (101 MHz, $CDCl_3$): δ = 164.34, 145.72, 135.23, 131.15, 130.12, 128.06, 126.29, 57.86, 36.29, 27.48, 24.00, 22.33, 21.78 ppm.

5. Synthesis of 3,3-dichloro-3,3a,4,6a-tetrahydro-2*H*-cyclopenta[*b*]furan-2-one (18)

A 10 mL pressure vial containing a magnetic stirring bar and capped with a septum was loaded with complex **1** (18.7 mg, 20 μmol). Air was expelled by three vacuum-argon cycles before hepta-1,6-dien-3-yle 2,2,2-trichloroacetate (**16**) (0.1030 g, 0.4 mmol), and dry and degassed toluene (2 mL) were added. The reaction mixture was heated for 1 h at 80 °C under stirring in a microwave reactor with a 150 W maximum power. After cooling to room temperature, the solvent was removed on a rotary evaporator. The residue was purified by column chromatography on silica gel (eluent: petroleum ether/ethyl acetate 98/2 to 90/10 v/v) to afford the pure product as a yellow oil. Yield: 40.2 mg (52%). ^{1}H NMR (400 MHz, CD_2Cl_2): δ = 6.24 (m, 1H, CH=CH), 5.94 (s, 1H, CH=CH), 5.44 (m, 1H, CH–O), 3.63 (m, 1H, CH–CCl_2), 2.84–2.76 (m, 1H, CH_2), 2.67–2.61 (m, 1H, CH_2) ppm. ^{13}C NMR (101 MHz, CD_2Cl_2): δ = 167.9 (s, C=O), 140.5 (s, =*C*H–CH), 126.6 (s, =*C*H–CH_2), 86.5 (s, CCl_2), 81.4 (s, CH–O), 54.2 (s, *C*H–CCl_2), 35.4 (s, CH_2) ppm.

References

1. Sauvage, X.; Borguet, Y.; Zaragoza, G.; Demonceau, A.; Delaude, L. *Adv. Synth. Catal.* **2009,** *351*, 441–455.
2. Quebatte, L.; Solari, E.; Scopelliti, R.; Severin, K. *Organometallics* **2005,** *24*, 1404–1406.
3. Overman, L. E. *J. Am. Chem. Soc.* **1976,** *98*, 2901–2910.
4. Seigal, B. A.; Fajardo, C.; Snapper, M. L. *J. Am. Chem. Soc.* **2005,** *127*, 16329–16332.
5. Singh, O. V.; Han, H. *Org. Lett.* **2006,** *6*, 3067–3070.
6. Edlin, C. D.; Faulkner, J.; Quayle, P. *Tetrahedron Lett.* **2006,** *47*, 1145–1151.
7. Nagashima, H.; Wakamatsu, H.; Ozaki, N.; Ishii, T.; Watanabe, M.; Tajima, T.; Itoh, K. *J. Org. Chem.* **1992,** *57*, 1682–1689.
