# Supplementary material for: Tandem catalysis of ring-closing metathesis/atom transfer radical reactions with homobimetallic ruthenium–arene complexes
Source: Beilstein J Org Chem. 2010 Dec 8;6:1167–73. doi: 10.3762/bjoc.6.133 (PMC3002019; doi:10.3762/bjoc.6.133)
Supplement: File 2 — Detailed crystallographic analysis of complex 7. [file Beilstein_J_Org_Chem-06-1167-s002.pdf]

Supporting Information

for

Tandem catalysis of ring-closing metathesis/ atom transfer radical reactions with homobimetallic ruthenium-arene complexes

Yannick Borguet[1], Xavier Sauvage[1], Guillermo Zaragoza[2], Albert Demonceau[1] and Lionel Delaude[*1]

Address:
[1]Laboratory of Macromolecular Chemistry and Organic Catalysis, Institut de Chimie (B6a), Université de Liège, Sart-Tilman par 4000 Liège, Belgium and
[2]Unidade de Raios X, Edificio CACTUS, Universidade de Santiago de Compostela, Campus Vida, 15782 Santiago de Compostela, Spain

Email:
Lionel Delaude - l.delaude@ulg.ac.be

* Corresponding author

Supporting Information File 2
Detailed crystallographic analysis of complex **7**

Table of content

1. X-Ray crystal struture determination S2-2

1.1. Crystal data for [(p-cymene)Ru(m-Cl)$_3$RuCl$_2$(PCy$_3$)] (7) S2-2

1.2. Data collection S2-2

1.3. Refinement S2-2

2. Detailed crystallographic analysis of complex **7** S2-3

References S2-4

1. X-Ray Crystal struture determination

Crystal data were collected on a Bruker APPEX II diffractometer using graphite-monochromated Mo-$K\alpha$ radiation (λ = 0.71073 Å) from a fine-focus sealed tube source at 100 K. Computing data and reduction was made with the APPEX II software [1]. The structure was solved using DIRDIF [2], and finally refined by full-matrix, least-squares based on F^2 by SHELXL [3]. An empirical absorption correction was applied using SADABS [4]. All nonhydrogen atoms were anisotropically refined and the hydrogen atom positions were calculated and refined by a riding model.

1.1. Crystal data for [(p-cymene)Ru(μ-Cl)$_3$RuCl$_2$(PCy$_3$)] (7)

$C_{28}H_{47}Cl_5P_1Ru_2$	F_{000} = 1612
M_r = 794.02	D_x = 1.59 Mg m^{-3}
monoclinic, $P2_1/c$	Mo $K\alpha$ radiation
Hall symbol: -P 2ybc	λ = 0.7107 Å
a = 19.6672 (13) Å	cell parameters from 1959 reflections
b = 16.8432 (12) Å,	θ = 2.4–26.4 °
c = 10.2146(6) Å	μ = 1.38 mm^{-1}
β = 101.481 (4) °	T = 100 K
V = 3316.0 (4) Å^3	red prism
Z = 4	0.19 × 0.14 × 0.02 mm

1.2. Data collection

BRUKER APPEX-II CCD diffractometer	6531 independent reflections
radiation source: fine-focus sealed tube	5077 reflections with $I > 2\sigma(I)$
T = 100 K	R_{int} = 0.065
ω and phi scans	θ_{max} = 26.0 °
absorption correction: multi-scan	θ_{min} = 1.6 °
BRUKER SADABS	h = −24→23
T_{min} = 0.865, T_{max} = 0.970	k = 0→20
71087 measured reflections	l = 0→12

1.3. Refinement

refinement on F^2	hydrogen site location: inferred from neighboring sites
least-squares matrix: full	H-atom parameters constrained
$R[F^2 > 2\sigma(F^2)]$ = 0.033	$w = 1/[\sigma^2(F_o^2) + (0.0093P)^2 + 6.5096P]$
$wR(F^2)$ = 0.062	where $P = (F_o^2 + 2F_c^2)/3$
S = 1.05	$(\Delta/\sigma)_{max}$ = 0.01
6531 reflections	$\Delta\rho_{max}$ = 0.54 e Å^{-3}
398 parameters	$\Delta\rho_{min}$ = −0.56 e Å^{-3}
96 restraints	extinction correction: none
secondary atom site location: difference Fourier map	primary atom site location: structure-invariant direct methods

2. Detailed crystallographic analysis of complex 7

In the crystal structure of complex **7** that we determined, there are three different molecular dispositions of the isopropyl substituent on the *p*-cymene ring with respect to the tricyclohexylphosphine ligand mixed in the unit cell (and the three opposite ones generated by the inversion center). One of them accounting for 26.7% of the total is characterized by a *cis* relationship, while the two other ones feature a *trans* disposition of the same groups and correspond, respectively, to 33.7% and 39.6% of the total (Figure S2-1). These percentages were calculated with free independent variables, and the sum of site occupation factors was restrained to 1. The refinement of the cyclohexyl group highlighted with an orange or blue hue was made with one free independent variable and the refined value is around 40%, which corresponds to the free variable value of one *trans* configuration. The *p*-cymene and cyclohexyl groups were then refined with the same free variable.

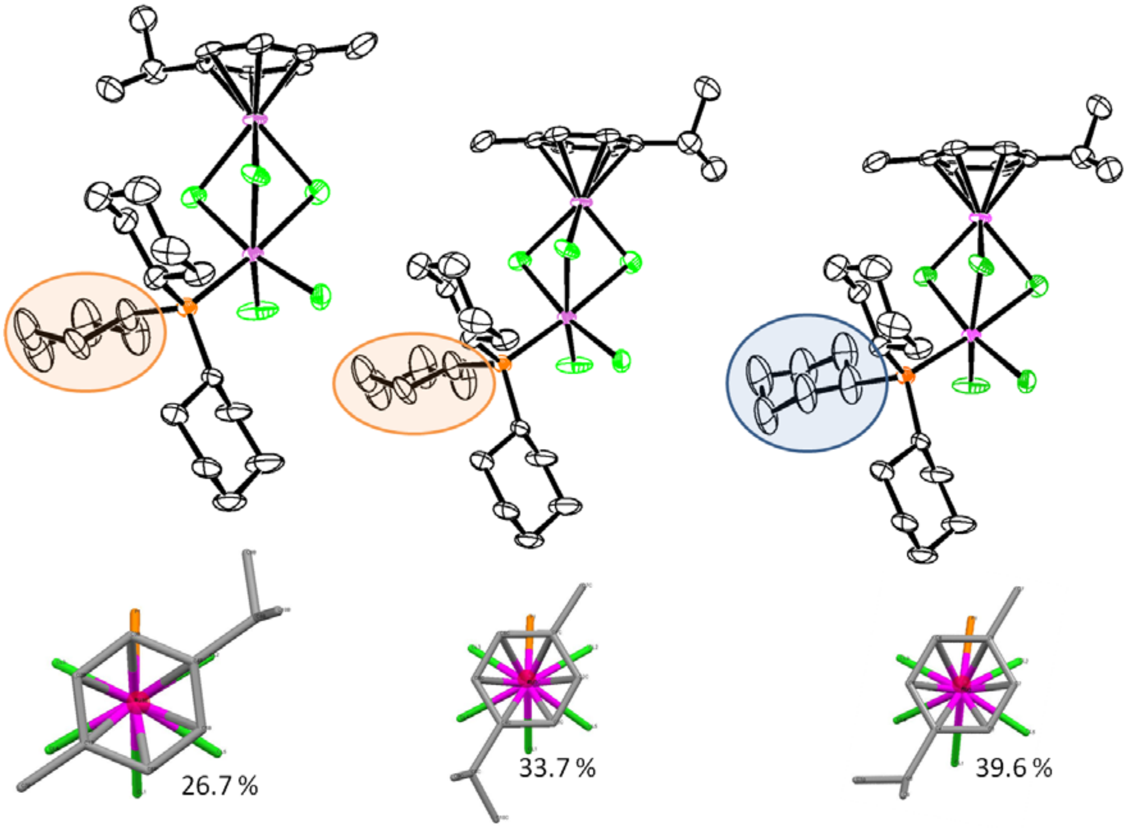

Figure S2-1.

Another difference between the three different configurations is the disposition around the aromatic ring and the angle P–Ru–(CH(CH_3)$_2$) in-plane projections (Figure S2-1).

In the molecular structure reported by Severin and co-workers for complex **7** [5], the isopropyl substituent on the *p*-cymene ring and the tricyclohexylphosphine ligand are *cis* to each other, as in one of our configurations, but the orientation relative to the plane angle P–Ru–($CH(CH_3)_2$) is different (Figure S2-2).

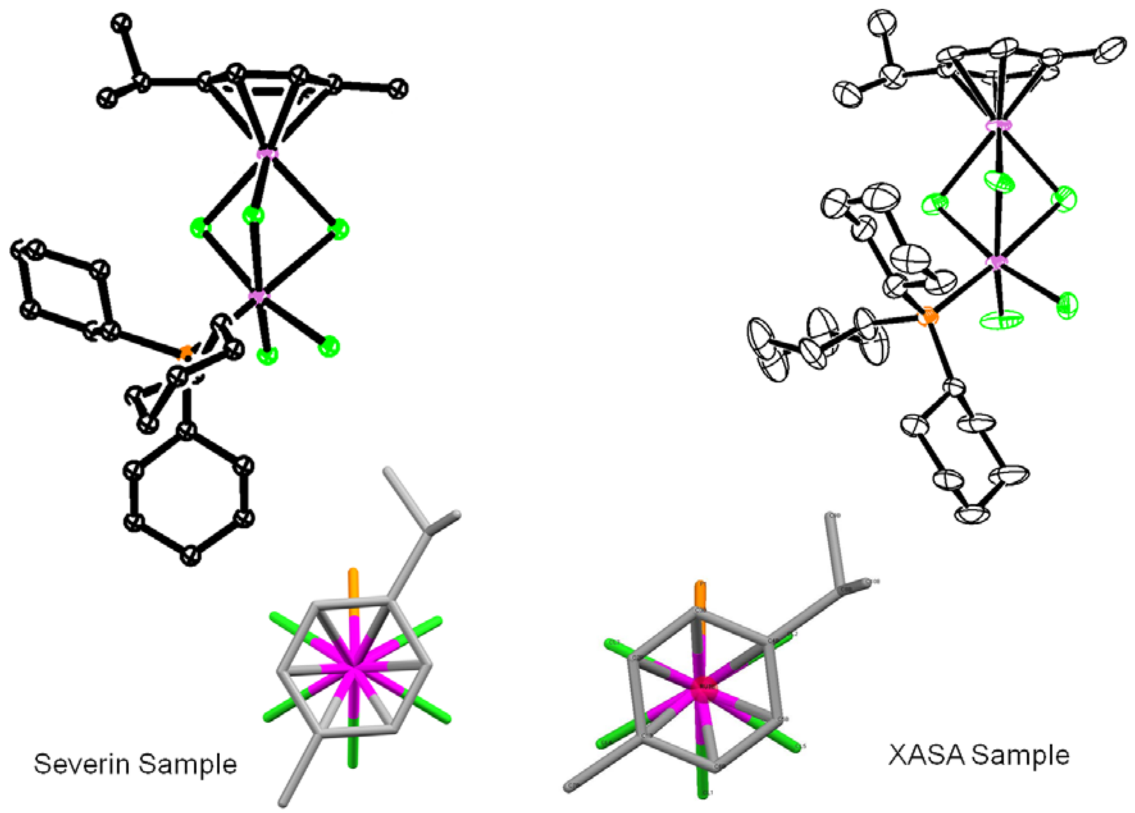

Figure S2-2.

References

1. Bruker *APPEX II, Bruker AXS Inc.*; Madison, WI, USA, 2004.
2. Beurskens, P. T.; Beurskens, G.; de Gelder, R.; Smits, J. M. M.; Garcia-Granda, S.; Gould, R. O. *DIRDIF-2007*; Radboud University: Nijmegen (The Netherlands), 2007.
3. Sheldrick, G. M. *SHELX-97 (SHELXS 97 and SHELXL 97), Programs for Crystal Structure Analyses*; University of Göttingen: Göttingen (Germany), 1998.
4. Sheldrick, G. M. *SADABS, Programs for Scaling and Correction of Area Detection Data*; University of Göttingen: Göttingen (Germany), 1996.
5. Quebatte, L.; Solari, E.; Scopelliti, R.; Severin, K. *Organometallics* **2005,** *24,* 1404–1406.
